# Differences Between Takotsubo and the Working Diagnosis of Myocardial Infarction With Nonobstructive Coronary Arteries

**DOI:** 10.3389/fcvm.2022.742010

**Published:** 2022-03-14

**Authors:** Javier Lopez-Pais, Bárbara Izquierdo Coronel, Sergio Raposeiras-Roubín, Leyre Álvarez Rodriguez, Oscar Vedia, Manuel Almendro-Delia, Alessandro Sionis, Agustin C. Martin-Garcia, Aitor Uribarri, Emilia Blanco, Irene Martín de Miguel, Emad Abu-Assi, David Galán Gil, Manuela Sestayo Fernández, Maria Jesús Espinosa Pascual, Rosa María Agra-Bermejo, Diego López Otero, Jose María García Acuña, Joaquín Jesús Alonso Martín, Jose Ramón Gonzalez-Juanatey, Miguel Ángel Perez de Juan Romero, Iván J. Núñez-Gil

**Affiliations:** ^1^Cardiology Department, Complexo Hospitalario Universitario de Ourense, Ourense, Spain; ^2^Cardiology Department, Hospital Universitario de Getafe, Madrid, Spain; ^3^Cardiology Department, Hospital Universitario Álvaro Cunqueiro, Vigo, Spain; ^4^Cardiology Department, Hospital Clínico Universitario de Santiago de Compostela, Santiago de Compostela, Spain; ^5^Cardiology Department, Hospital Clínico San Carlos, Madrid, Spain; ^6^Cardiology Department, Hospital Universitario Virgen Macarena, Sevilla, Spain; ^7^Cardiology Department, Hospital de Sant Pau, Barcelona, Spain; ^8^Cardiology Department, Hospital Clínico Universitario de Salamanca, Salamanca, Spain; ^9^Cardiology Department, Hospital Clínico Universitario de Valladolid, Valladolid, Spain; ^10^Cardiology Department, Hospital Arnau de Vilanova, Lérida, Spain; ^11^Cardiology Department, Hospital General Universitario Gregorio Marañón, Madrid, Spain

**Keywords:** Takotsubo, MINOCA, definition, prognosis, working diagnosis

## Abstract

**Aim:**

Whether Takotsubo syndrome (TTS) should be classified within myocardial infarction with non-obstructive coronary arteries (MINOCAs) is still controversial. The aim of this work was to evaluate the main differences between TTS and non-TTS MINOCAs.

**Methods and Results:**

A cohort study based on two prospective registries: TTS from the RETAKO registry (*N*:1,015) and patients with non-TTS MINOCAs from contemporary records of acute myocardial infarction from five 5 national centers (*N*:1,080). Definitions and management recommended by the ESC were used. Survival analysis was based on the Cox regression analysis; propensity score matching (PS) was created to adjust prognostic variables. Takotsubo syndrome were more often women (85.9 vs. 51.9%; *p* < 0.001) and older (69.4 ± 12.5 vs. 64.5 ± 14.1 years; *p* < 0.001). Atrial fibrillation (AF) was more frequent in non-TTS MINOCAs (10.4 vs. 14.4%; *p* = 0.007). Psychiatric disorders were more prevalent in TTS (15.5 vs. 10.2%, *p* < 0.001). In-hospital mortality and complications were higher in TTS: 3.4 vs. 1.8%, (*p* = 0.015), and 25.8 vs. 11.5%, (*p* < 0.001). Global mortality before PS matching was 16.1% in non-TTS MINOCAs and 8.1% in TTS. Median follow-up was 32.4 months; after PS matching, TTS had fewer major adverse cardiovascular events (MACEs): hazard ratio (HR) 0.59; 95% CI 0.42–0.83. There were no differences in global mortality (HR 0.87; CI: 0.64–1.19), but TTS had lower cardiovascular mortality (HR 0.58; CI: 0.35–0.98).

**Conclusion:**

Compared to the rest of MINOCAs, TTS presents a different patient profile and a more aggressive acute phase. However, its long-term cardiovascular prognosis is better. These results support that TTS should be considered a separate entity with unique characteristics and prognosis.

## Key Points

– **What is already known about this subject?**
  Takotsubo syndrome may be considered as acute cardiomyopathy, although it can mimic an acute coronary syndrome. For this reason, it first was included in the working diagnosis of MINOCAs.
– **What does this study add?**
  Takotsubo syndrome has a different clinical profile that MINOCAs, with a more aggressive acute phase regardless of what its long-term cardiovascular prognosis is better as compared with the rest of patients with MINOCAs.

## Introduction

Medicine has never been an exact science. To minimize errors caused by this inevitable fact, many classifications have been developed to treat diseases as precisely as possible (1). However, there are still some gaps in evidence, even in deeply studied fields such as acute myocardial infarction (AMI) (2). In the recent decades, scientific societies have developed guidelines to act as a consensus to standardize best clinical practice (3). Guidelines must be clear so that physicians can offer patients the best care available. Despite having its chapter in guidelines, myocardial infarction with nonobstructive coronary arteries (MINOCAs) remains an unclear subject (4–9). This is due to two main facts. At first, many physiopathological mechanisms are underlying the blurred concept of MINOCAs. Second, MINOCAs being a “*working diagnosis”* (4) leads to the paradox that the same disease could be a MINOCAs or not depending on the time of diagnosis.

Myocardial infarction with nonobstructive coronary arteries represent a considerable percentage of all the AMI (5–14% of all the AMI undergoing coronary angiogram) (4, 5, 10, 11). Recent publications suggest that MINOCAs prognosis is worse than previously considered, with first-year mortality reaching up to 5% (12) and the proportion of MACE around 20% (9). One of the traditional mechanisms of MINOCAs was Takotsubo syndrome (TTS) (1, 4, 11, 13–15), a transient ventricular dysfunction that mimics an AMI in the absence of coronary obstruction (16, 17). TTS pathophysiology remains unclear. However, with the available evidence, it seems that dysregulation in sympathetic neurohormonal axis plays a vital role, leading, somehow, to endothelial dysfunction and vasospasm (18–20). This hypothesis suggests that even in the acute phase, TTS is a phenocopy of an AMI, its notion may be closer to an acute myocardiopathy than to an AMI. The 2016 position paper from ESC includes the TTS in the definition of MINOCAs (4), whereas 2018 4^th^ Universal Definition of Infarction (1) and later scientific statements from AHA considers the TTS as a separate entity (20).

This study was developed based on that idea, with the objective of comparing TTS to the rest of patients with MINOCAs, searching for differences between them in clinical profile and mid-term prognosis.

## Methods

Retrospective study of two cohorts based on prospective registries of patients with TTS and AMI, respectively, with the objective of comparing a TTS with a non-TTS MINOCAs cohort.

### Study Population

Takotsubo Syndrome cohort was based on the National Multicenter Registry on TTS (RETAKO), sponsored by the Section of Ischemic Heart Disease and Cardiovascular Acute Care of the Spanish Society of Cardiology. It is a prospective and voluntary study, including 24 centers in the country. Its rationale and design have been previously described (14), and in summary, it prospectively includes all consecutive patients with TTS who fulfill the modified Mayo Clinic diagnostic criteria (13): (a) transient left ventricular dysfunction with apical, midventricular or basal segmental alterations extending beyond the territory supplied by a single coronary artery; (b) absence of significant obstructive coronary artery disease (luminal narrowing >50%) or angiographic evidence of a complicated atheroma plaque; (c) new electrocardiogram (EKG) changes (ST-segment elevation and/or negative T-waves) or moderate elevation of cardiac troponins; (d) absence of myocarditis or pheochromocytoma (13, 14). Complete normalization of wall motion abnormalities and left ventricular ejection fraction (LVEF) was required, except in the event of death. For this analysis, 1,055 subjects with TTS were consecutively included between January 1, 2003, and December 31, 2017. The closing date to follow-up was settled on September 30, 2018.

The non-TTS MINOCAs cohort was created with all consecutive patients admitted for AMI in five national centers during the same period (2003–2017, follow-up settled on September 2018). After excluding patients with TTS, 1,080 patients were classified as MINOCAs according to the ESC Position Paper on MINOCAs (4): AMI according to the 3rd Universal Definition of Infarction (which equals to type 1 MI in the 4th Universal Definition of Infarction) (1, 21); and coronary arteries without significant angiographic obstruction (meaning <50% of stenosis). In order to obtain a working diagnosis for MINOCAs, there could not be any other obvious explanation for the event at the moment of its presentation; this fact was confirmed in every case after a thorough review carried out by trained cardiology staff.

Patients referred for coronary angiography only to rule out coronary disease in the context of another entity, were not included in the study (pulmonary emboli, aortic stenosis, hypertrophic myocardiopathy, myocarditis). Patients initially treated and managed as an AMI, but that later happened to have myocarditis confirmed by magnetic resonance, were not classified as MINOCAs, following the 4th Universal Definition of Infarction. Patients who fitted the new definition of acute myocardial damage (1) were excluded.

Standardized forms were used for the setting-up of the database, including demographical information, epidemiological data, drugs received during hospital admission and at discharge attending to criteria of the physician, and other relevant clinical information. This study considered the presence of AF both when it was diagnosed during hospitalization and as a prior antecedent of this arrhythmia. Psychiatric diagnoses were considered, according to the Diagnostic and Statistical Manual of Mental Disorders, fifth edition (22). All in-hospital complications and in-hospital mortality were registered.

### Follow-Up and Outcomes

Follow-up analysis included: MACE (a composite of a recurrence of AMI, transient ischemic attack (TIA)/stroke, or death from cardiovascular cause), time to first readmission from cardiovascular causes, cardiovascular mortality (caused by myocardial infarction, sudden death, heart failure, or other vascular causes) and death from any cause. Follow-up data were collected *via* clinical visits, medical records, institutional databases, or telephone interviews. Data was censored at 8 years. If a possible prespecified outcome was observed, a review of electronic medical records by experienced local investigators was mandatory for event adjudication. This study was approved by the institutional review board and followed the tenets of the Declaration of Helsinki.

### Statistical Analysis

Statistical analysis was performed with the IBM SPSS 24.0 software (SPSS Incorporation, Chicago, Illinois, USA) with the essentials for R-3.2 package and the propensity score (PS) matching patch 3.04. Continuous variables are presented as means and SDs or as medians with SD or interquartile range, as appropriate. Categorical variables are provided with percentages. Pearson chi-square or Student *t*-tests and their nonparametric equivalent were used depending on the variable type. A PS was created to adjust for baseline differences between subjects with and patients with non-TTS MINOCAs (Supplementary Table 1). A logistic regression model with TTS as outcome was used to generate PSs for all the subjects, using a bond 1:1 matching with a caliper of 0.01. Variables that are main potential risk factors for cardiovascular events (age, hypertension, diabetes, tobacco, and dyslipidemia) were included in the model. Time to event of the primary outcomes was analyzed with Cox regression analysis using the 1:1 matching based on the PS. Odds ratios and Hazard ratios (HRs) are reported with 95% CIs. A two-sided *P* value of <0.05 was considered statistically significant.

## Results

### Baseline Comparison

Patients with TTS, as compared with non-TTS MINOCAs, were more frequently women (*p* < 0.001), older (69.4 ± 12.5 vs. 64.5 ± 14.1; *p* < 0.001), with a higher prevalence of hypertension and no significant differences regarding the rest of cardiovascular risk factors. Despite that, AF was more frequent in non-TTS MINOCAs (10.4 vs. 14.4%; *p* = 0.007). Psychiatric disorders were more prevalent in individuals with TTS (15.5 vs. 10.2%, *p* < 0.001). During hospitalization because of other causes, TTS was an intercurrent complication in 22.4% of the cases as compared with 0.8% in the non-TTS group (*p* < 0.001). Details of baseline characteristics are summarized in Table 1.

**Table 1:** Baseline characteristics.

**Variables**	**Takotsubo patients** ***N*****:1,015**	**Non-takotsubo MINOCA patients** ***N*****:1,080**	***P*** **-value**
**Age**	69.4 ± 12.5	64.5 ± 14.1	<0.001
**Female sex**	872/1,015 (85.9%)	561/1,080 (51.9%)	<0.001
**Cardiovascular risk factors**			
Smoking	259/1,015 (25.5%)	274/1,080 (25.4%)	0.9
Diabetes	181/1,015 (17.8%)	186/1,080 (17.2%)	0.7
Dyslipidemia	449/1,015 (44.2%)	516/1,080 (47.8%)	0.1
Hypertension	654/1,015 (64.4%)	645/1,080 (59.7%)	0.02
**Cardiovascular history**			
ACVA	53/1,015 (5.2%)	63/1,080 (5.8%)	0.5
AF or flutter	106/1,015 (10.4%)	155/1,080 (14.4%)	0.007
PVD	42/1,015 (4.1%)	49/1,080 (4.5%)	0.6
**Comorbidities**			
Chronic kidney disease	66/1,015 (6.5%)	85/1,080 (7.9%)	0.2
OSAHS	29/1,015 (2.9%)	27/1,080 (2.5%)	0.6
Active cancer	113/1,015 (11.1%)	107/1,080 (9.9%)	0.3
Autoimmune disease	67/1,015 (6.6%)	64/1,080 (5.9%)	0.5
Connective tissue disease	12/1,015 (1.2%)	10/1,080 (0.9%)	0.5
Psychiatric illness	157/1,015 (15.5%)	110/1,080 (10.2%)	<0.001
Migraine	34/1,015 (3.3%)	9/1,080 (0.8%)	<0.001
Intercurrent complication^*^	227/1,015 (22.4%)	9/1,080 (0.8%)	<0.001
**Cocaine abuse**	5/1015 (0.5%)	5/1080 (0.5%)	0.9

*Values are mean ± SD or n (%)*.

*ACVA, acute cerebrovascular accident; AF, atrial fibrillation; PVD, peripheral vascular disease; OSAHS, obstructive sleep apnea/hypopnea syndrome; MINOCA, myocardial infarction with nonobstructive coronary arteries*.

* *While hospitalization for another cause*.

### Clinical Presentation

At presentation, 19.8% of patients with TTS arrived on Killip–Kimball class III or IV as compared with 3.5% in the non-TTS (*p* < 0.001). Heart rate in individuals with TTS was higher, and their systolic blood pressure was lower. Abnormal EKG was present in 77.3% of patients with TTS as compared with 32.2% of non-TTS (*p* < 0.001). As Table 2 shows, TTS individuals had higher levels of troponin, natriuretic peptides, and C-reactive protein. Hemoglobin was lower in patients with TTS (13.2 ± 2.3 vs. 13.9 ± 4.5, g/decilitre; *p* < 0.001). The median ejection fraction of patients with TTS was 42.1 ± 13.1% as compared with 56.1 ± 10.2% of non-TTS (*p* < 0.001). Ventricular dysfunction was more than twofold present in the TTS group than in non-TTS (*p* < 0.001). Complications during hospitalization (infarction, pulmonary edema, shock, stroke, hemorrhages, mechanical complications) occurred in 25.8% of patients with TTS as compared with 11.5% of non-TTS (*p* < 0.001). In-hospital mortality was higher in patients with TTS (3.4 vs. 1.8%, *p* = 0.015). Details of the analysis are provided in Table 3.

**Table 2:** Main clinical data and test findings.

**Variables**	**Takotsubo patients** ***N*****:1,015**	**Non-takotsubo MINOCA patients** ***N*****:1,080**	***P*** **-value**
**Data at admission**			
Killip-Kimball III or IV	209/1,055 (19.8%)	38/1,080 (3.5%)	<0.001
Heart rate	83.99 ± 14.66	80.67 ± 24.30	0.001
Systolic blood pressure (mmHg)	128.12 ± 29	137.76 ± 28.16	<0.001
**Laboratory**			
Troponine T (ng/L)	485.00 (94.25–946.75)	3.74 (0.46–25.42)	<0.001
Troponine I (ng/ml)	2.7 (1.05–5.60)	0.50 (0.12–3.46)	0.02
CK (U/L)	62.0 (15.4–236.5)	184.0 (104.5–412.0)	0.75
Haemoglobine (g/dL)	13.17 ± 2.26	13.90 ± 4.52	0.001
Leucocytes	10.64 ± 4.61	10.31 ± 29.58	0.81
C-reactive protein (mg/L)	12.00 (3.00–54.5.00)	3.00 (0.57–8.00)	0.02
NT-proBNP (pg/mL)	3,600.0 (1,621.0–9,001.5)	1,241.0 (386.5–2,848.0)	<0.001
Creatinine (mg/dL)	1.09 ± 0.83	1 ± 0.62	0.03
**Electrocardiogram**			
Abnormal EKG	785/1,015 (77.3%)	348/1,080 (32.2%)	<0.001
ST segment elevation	517/1,015 (50.9%)	85/1,080 (7.9%)	<0.001
Non-ST segment elevation	138/1,015 (13.6%)	91/1,080 (8.4%)	<0.001
Negative T waves	333/1,015 (32.8%)	126/1,080 (11.7%)	<0.001
**Echocardiogram**			
Left ventricular ejection fraction	42.1 ± 13.11	56.09 ± 10.20	<0.001
Left ventricular dysfunction	747/1,015 (73.6%)	308/1,080 (28.5%)	<0.001
Moderate to severe valvulopathy	313/1,015 (30.8%)	196/1,080 (18.1%)	<0.001
Pulmonary hypertension	158/1015 (15.6%)	13/1080 (1.2%)	<0.001
**MRI performed**	266/1,015 (26.2%)	56/1,080 (5.2%)	<0.001
**Days of hospitalization**	6.0 (4.0–10.0)	7.0 (5.0–12.0)	<0.001

*Values are mean ± SD, median (P25-P75), or n (%). Hear rate expressed in beats per minute*.

*MINOCA, myocardial infarction with nonobstructive coronary arteries*.

**Table 3:** Complications during hospitalization.

**Variables**	**Takotsubo patients** ***N*****:1,015**	**Non-takotsubo MINOCA patients** ***N*****:1,080**	***P*** **-value**
Total	262/1,015 (25.8%)	124/1,080 (11.5%)	<0.001
Acute pulmonary edema	217/1,015 (21.4%)	45/1,080 (4.2%)	<0.001
Cardiogenic shock	121/1,015 (11.9%)	31/1,080 (2.9%)	<0.001
Reinfarction	4/1,015 (0.4%)	7/1,080 (0.6%)	0.4
Major bleeding event	35/1,015 (3.4%)	56/1,080 (5.2%)	0.05
ACVA	27/1,015 (2.7%)	8/1,080 (0.7%)	0.001
Recovered cardiac arrest	5/1,015 (0.5%)	9/1,080 (0.8%)	0.4
Mechanical complications	3/1,015 (0.3%)	1/1,080 (0.1%)	0.3
In-hospital mortality	35/1,015 (3.4%)	19/1,080 (1.8%)	0.015

*ACVA, acute cerebrovascular accident*.

### Treatment

Regarding treatment at discharge (Table 4), dual antiplatelet therapy (DAT) was prescribed in 1.0% of patients with TTS as compared with 23.5% of non-TTS MINOCAs (*p* < 0.001), beta-blocker prescription and angiotensin-converting enzyme inhibitors/angiotensin II receptor blockers (ACEI/ARB) were higher in the TTS cohort (58.3 vs. 52.8%; *p* = 0.011 and 59.5 vs. 50.0%; *p* < 0.001, respectively). The prescription of statins was lower in individuals with TTS (49.6 vs. 76.1%; *p* < 0.001).

**Table 4:** Treatment at discharge.

**Variables**	**Takotsubo patients** ***N*****:1,015**	**Non-takotsubo MINOCA patients** ***N*****:1,080**	***P*** **-value**
Acetyl salicylic acid	48/1,015 (4.7%)	733/1,080 (67.9%)	<0.001
Dual antiplatelt therapy	10/1,015 (1.0%)	254/1,080 (23.5%)	<0.001
Anticoagulants	173/1,015 (17.0%)	186/1,080 (17.2%)	0.9
Beta-blockers	592/1,015 (58.3%)	570/1,080 (52.8%)	0.01
ACE inhibitors/ARB	604/1,015 (59.5%)	540/1,080 (50.0%)	<0.001
Nitrates	42/1,015 (4.1%)	10/1,080 (0.9%)	<0.001
Diuretics	233/1,015 (23.0%)	90/1,080 (8.3%)	<0.001
Calcium antagonists	90/1,015 (8.9%)	148/1,080 (13.7%)	<0.001
Statins	503/1,015 (49.6%)	822/1,080 (76.1%)	<0.001

*MINOCAs, myocardial infarction with nonobstructive coronary arteries; ACEI/ARB, angiotensin-converting enzyme inhibitor/angiotensin receptor blocker*.

### Prognosis Based on Propensity Score Analysis

Median follow-up was 32.4 months [percentile (P) 25: 3.8 months, *P* = 75: 50.1 months]; survival analysis was performed on the model created with a PS matching to adjust for baseline differences in variables that are potential arguments for cardiovascular events (age, diabetes, tobacco, hypertension, and dyslipidemia). After a bond 1:1 matching (with a caliper of 0.01), 745 patients remained in each cohort.

MACE (cardiovascular mortality, infarction or TIA/stroke) were lower in patients with TTS [hazard ratio (HR) 0.59; 95% CI 0.42 to 0.83]. Individually, both the composite outcomes CV mortality and TIA/stroke were lower in patients with TTS and, AMI had a nonsignificant reduction in patients with TTS. Although global mortality was 16.1% in non-TTS MINOCAs and 8.1% in TTS, there were no statistical differences in global mortality after PS matching.

**Figure 1:**
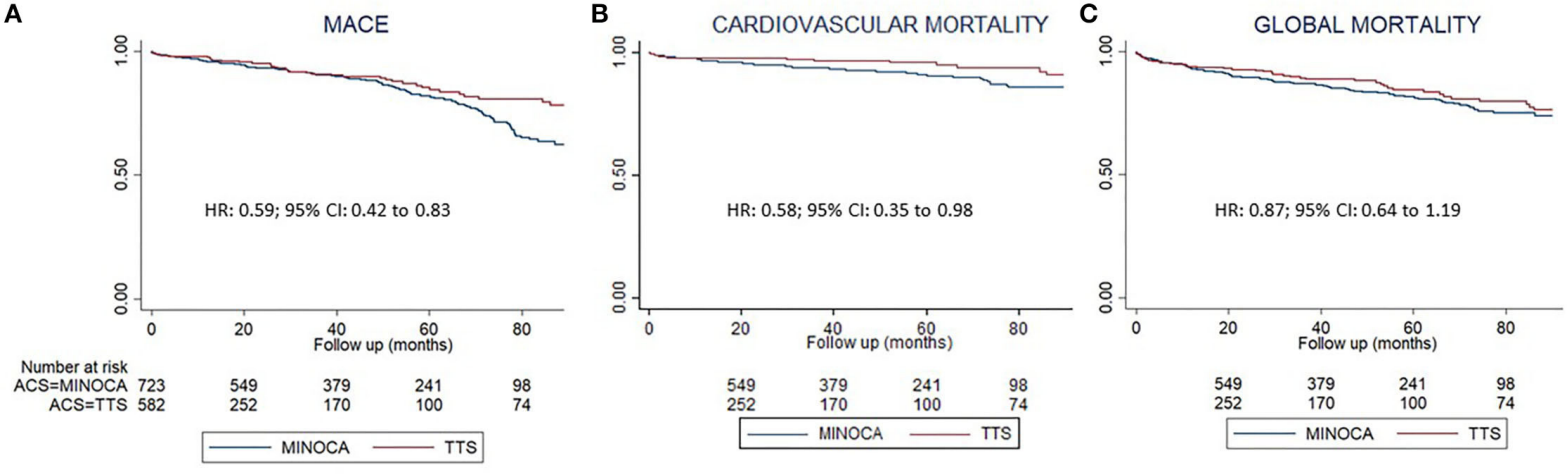
Follow-up analysis based on a propensity score matching. Kaplan Meier representation of the survival analysis, on them, Cox regression results. In red nontakotsubo MINOCA, in blue Takotsubo syndrome patients. **(A)** Major adverse cardiovascular events (MACE). **(B)** Cardiovascular mortality. **(C)** All-cause mortality. HR, hazard ratio.

Cardiovascular readmissions were higher in non-TTS individuals (HR: 0.58, CI: 0.42–0.81). The time-to-event analysis is shown in Figure 1 and Table 5.

**Table 5:** Follow-up survival analysis.

	**Takotsubo MINOCA**	**Non-Takotsubo Syndrome**	**HR**	**C.I. 95%**	***P*** **-value**
MACE (%)	6.3	17.4	0.59	0.42–0.83	0.002
CV Mortality (%)	2.7	7.8	0.58	0.35–0.98	0.042
Infarction (%)	2.4	6.8	0.60	1.34–1.02	0.060
TIA or Stroke (%)	1.1	4.6	0.44	0.21–0.96	0.039
CV Re-admission (%)	6.6	18.1	0.58	0.42–0.81	0.001
Global Mortality (%)	8.1	16.1	0.87	0.64–1.19	0.392

*Follow-up survival analysis adjusted by propensity score matching with main potential arguments of cardiovascular events (age, hypertension, diabetes, tobacco, and dyslipidemia)*.

*MINOCAs, myocardial infarction with nonobstructive coronary arteries; MACE, major adverse cardiovascular events (infarction, transient ischemic attack/stroke, or cardiovascular death); CV, cardiovascular; TIA, transient ischemic attack; HR, hazard ratio*.

## Discussion

The main conclusion of this study, based on large multicenter registries, is that TTS has a different clinical profile than the rest of MINOCAs: (1) TTS affects women at a greater rate; (2) acute phase of TTS is much more aggressive, with worst Killip–Kimball class, more EKG abnormalities, higher levels of cardiac biomarkers and higher in-hospital mortality; and (3) Long-term cardiovascular prognosis of TTS is better, with less MACE.

This study is the first large multicenter study to compare MINOCAs and non-TTS MINOCAs. The main clinical implications of these results are its implications on follow-up management after discharge. Once the acute phase has been managed, this article might suggest that patients with TTS can safely be transferred to the general practitioner, whereas non-TTS MINOCAs are still at considerable risk of MACE and could benefit from specific follow-up.

A higher rate of AF in patients with non-TTS MINOCAs is remarkable, even when considering they are younger and with less hypertension than patients with TTS. The association of AF with MINOCAs could be explained as emboli being the underlying physiopathological mechanism for the event (1, 4, 5).

Another interesting fact is the higher proportion of migraine in the group of TTS. Nociceptive mechanisms of migraine are thought to be related to vascular tone dysregulation (23), one of the possible mechanisms that partake in TTS.

The association of psychiatric disorders with TTS is well-established (13, 20); these results being consistent with the previous literature. In the recent years, TTS physical triggers are acquiring more relevance (24), and this study reflects how TTS patients have more extracardiac comorbidities, with lower hemoglobin levels and higher C-reactive protein. The research of Santoro et al. (25) has already exposed the differences in inflammatory interleukins patter between TTS and acute coronary syndrome. Patients with TTS showed increased levels of anti-inflammatory interleukins during the acute phase.

Another key issue is that TTS was an intercurrent complication in hospitalization due to other causes in almost thirtyfold over patients with non-TTS.

The acute phase of TTS is aggressive compared to non-TTS MINOCAs, with twofold in-hospital mortality. As RETAKO investigators have previously described, cardiogenic shock is not unusual in patients with non-TTS (12%) (26), almost fourfold the proportion of cardiogenic shock in non-TTS MINOCAs. Recently, an interesting score has been proposed to assess the risk of in-hospital complications on TTS (27).

The significant events during follow-up in the MINOCAs group could be due to misdiagnosis at index episode. This study shows a low use of cardiac magnetic resonance (CMR) in accordance with Bhatia et al. (28), who proposed that CMR should be mandatory in every patient with elevated troponin and normal coronary angiography. Recently, the group of Dastidar has shown the prognostic implications of the use of CMR in this context (29).

Although the pathophysiology of TTS remains unknown, the evidence available supports chronic treatment with ACEI/ARB (30, 31), despite the limitation of lack of randomized control trials (first one is currently under recruitment [MINOCAs-BAT: EudraCT number: 2018-000889-11, ClinicalTrials.gov identifier: NCT03686696]). However, this study reflects that in real life, only 58% of the patients were treated according to the guidelines. Even lower rates (23.5%) are registered in the non-TTS MINOCAs group for the DAT, despite ESC recommendations.

Going back to the starting point, it is still unclear how clinicians should deal with this disarrayed group of patients. Artificial intelligence is meant to be the next revolution in medicine, but it is also based on proper and precise classifications (32) that need to be reinforced by evidence.

In conclusion, according to this study, TTS shows clear differences from the rest of the MINOCAs group. TTS presents profile of a unique patient, a more aggressive clinical presentation, and worse in-hospital outcomes. However, their long-term cardiovascular prognosis is better than non-TTS MINOCAs.

Based on this, it is congruent to propose that TTS should be considered acute cardiomyopathy. Although it can mimic an acute coronary syndrome, once the diagnosis of TTS is made, including it on the working diagnosis MINOCAs do not offer any advantage. This work supports the recent exclusion of TTS from MINOCAs.

### Study Limitations

Because of its observational nature, unmeasured confounders could constrain causal inference in this study. However, prospective follow-up may allow the application of the causal inference methods.

As MINOCAs is a syndromic diagnosis rather than a physiopathological one, this could lead to disagreement between physicians at the time of considering whether a patient is a MINOCAs or not. This fact is mitigated by well-delimiting the inclusion criteria, the thorough review conducted by the authors, and the multicentric nature of the study.

## Conclusion

**Figure 2:**
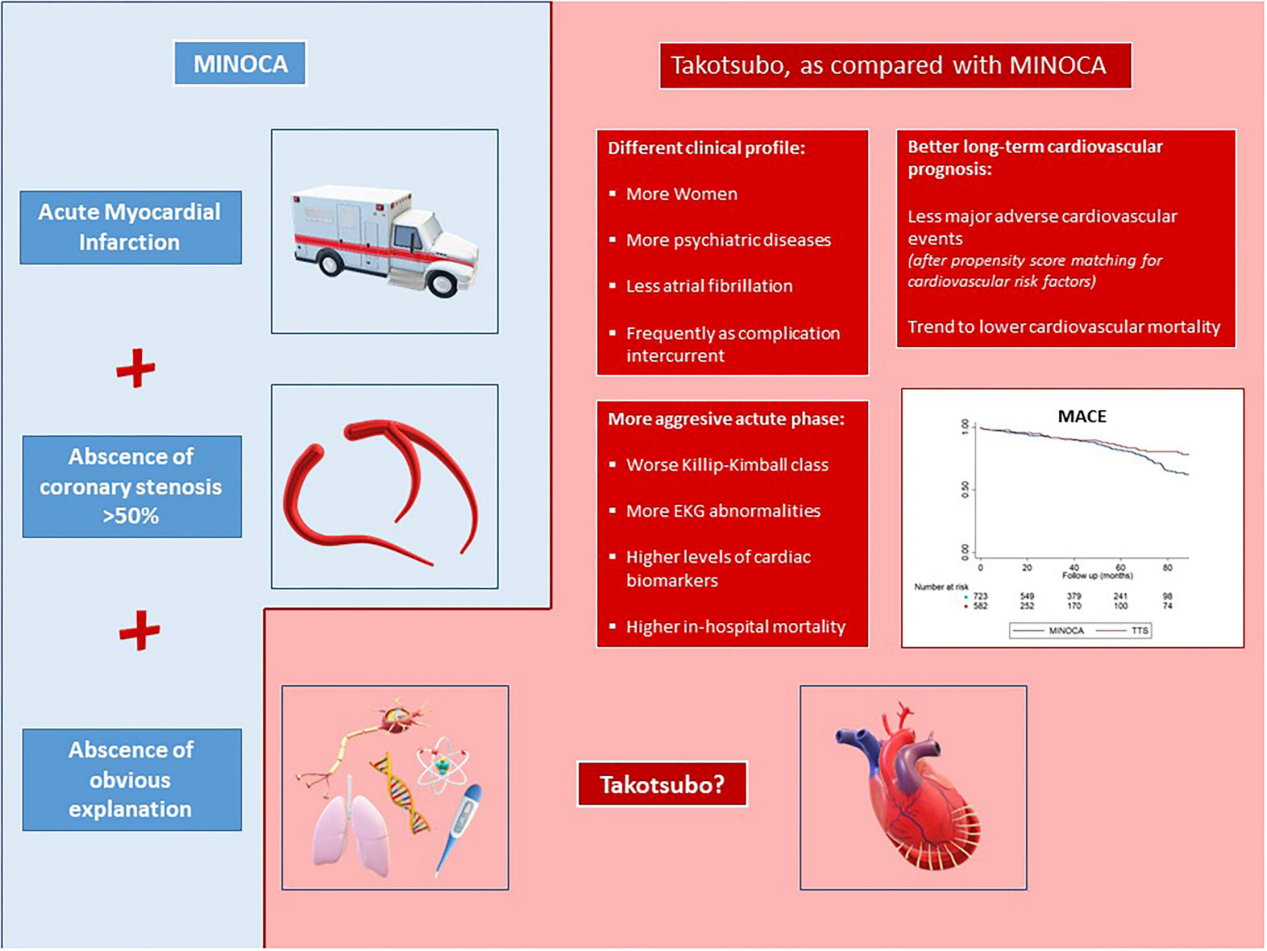
Takotsubo shows clear differences with patients with MINOCAs, with a unique profile of the patient, a more aggressive acute phase and better long-term cardiovascular prognosis. MINOCAs, myocardial infarction with nonobstructive coronary arteries; TTS, Takotsubo syndrome; MACE, major adverse cardiovascular events (infarction, transient ischemic attack/stroke, or cardiovascular death); CV, cardiovascular; TIA, transient ischemic attack; HR, hazard ratio.

Takotsubo syndrome has a different clinical profile than the rest of MINOCAs (Figure 2):

(1) Takotsubo syndrome affects women to a greater extent; they have more psychiatric diseases and less AF. TTS is most frequently an intercurrent complication during hospitalization for other causes.
(2) The acute phase of TTS is much more aggressive, with worst Killip–Kimball class, more EKG abnormalities, higher levels of cardiac biomarkers, and higher in-hospital mortality.
(3) The long-term cardiovascular prognosis of TTS is better, with less MACE.

## Data Availability Statement

The original contributions presented in the study are included in the article/Supplementary Material, further inquiries can be directed to the corresponding author.

## Ethics Statement

The studies involving human participants were reviewed and approved by Comité Ético de Galicia. The patients/participants provided their written informed consent to participate in this study.

## Author Contributions

JL-P and IN-G contributed to the conception or design of the work and final approval of the version to be published. BI, SR-R, MA-D, LÁ, OV, AS, AM-G, AU, EB, IM, EA-A, DG, MS, ME, and RA-B contributed to the data collection. JL-P, MA-D, DL, JG, JA, and IN-G contributed to the data analysis and interpretation. JL-P and BI drafted the article. DL, JG, JA, JG-J, MP, and IN-G contributed to the critical revision of the article. All authors contributed to the article and approved the submitted version.

## Funding

The RETAKO registry was sponsored by the Spanish Society of Cardiology.

## Conflict of Interest

The authors declare that the research was conducted in the absence of any commercial or financial relationships that could be construed as a potential conflict of interest. The reviewer RS-R declared a shared affiliation with one of the authors, IM to the handling editor at time of review.

## Publisher's Note

All claims expressed in this article are solely those of the authors and do not necessarily represent those of their affiliated organizations, or those of the publisher, the editors and the reviewers. Any product that may be evaluated in this article, or claim that may be made by its manufacturer, is not guaranteed or endorsed by the publisher.
