# Supplementary material for: Differences Between Takotsubo and the Working Diagnosis of Myocardial Infarction With Nonobstructive Coronary Arteries
Source: Front Cardiovasc Med. 2022 Mar 14;9:742010. doi: 10.3389/fcvm.2022.742010 (PMC8964136; doi:10.3389/fcvm.2022.742010)
Supplement: Supplementary file 1 [file Table_1.DOCX]

| Table supplementary 1: Baseline characteristics after propensity score matching according to the main potential arguments of cardiovascular events (age, hypertension, diabetes, tobacco, and dyslipidemia) | | | |
| --- | --- | --- | --- |
| Baseline characteristics | **Takotsubo patients**  **N:745** | **Non-takotsubo MINOCA patients**  **N:745** | **P-value** |
| Age | 68.8±11.6 | 68.4±11.9 | 0.567 |
| Female sex | 651/745 (87.4%) | 413/745 (55.4%) | <0.001 |
| Cardiovascular risk factors |  |  |  |
| *Smoking* | 150/745 (20.1%) | 173/745 (23.2%) | 0.148 |
| *Diabetes* | 119/745 (16.0%) | 141/745 (18.9%) | 0.133 |
| *Dyslipidemia* | 343/745 (46.0%) | 339/745 (45.5%) | 0.835 |
| *Hypertension* | 485/745 (65.1%) | 481/745 (64.6%) | 0.828 |
| Vascular history |  |  |  |
| *ACVA* | 36/745 (4.8%) | 50/745 (6.7%) | 0.120 |
| *PVD* | 42/1015 (4.1%) | 28/610 (4.6%) | 0.664 |
| Other comorbidities |  |  |  |
| *Chronic kidney disease* | 46/745 (6.2%) | 64/745 (8.6%) | 0.075 |
| *OSAHS* | 17/745 (2.3%) | 22/745 (3.0%) | 0.417 |
| *Active cancer* | 87/745 (11.7%) | 88/745 (11.8%) | 0.936 |
| *Autoimmune disease* | 51/745 (6.8%) | 40/745 (5.4%) | 0.234 |
| *Connective tissue disease* | 11/745 (1.5%) | 7/745 (0.9%) | 0.343 |
| *Psychiatric illness* | 123/745 (16.5%) | 67/745 (9.0%) | <0.001 |
| ACVA: Acute cerebrovascular accident. AF: Atrial fibrillation. PVD: Peripheral vascular disease. OSAHS: Obstructive sleep apnoea/hypopnoea syndrome. MINOCA: Myocardial infarction with non-obstructive coronary arteries.  *While hospitalisation for another cause | | | |
